# Cord-like omental entanglement causing right ovarian torsion in an octogenarian woman: a case report

**DOI:** 10.1093/jscr/rjag617

**Published:** 2026-07-21

**Authors:** Takahiro Inoue, Kyouichi Uemura

**Affiliations:** Department of Gastrointestinal Surgery, Sapporo Kojinkai Memorial Hospital, Kita 1-jo Nishi 15-chome, Nishi-ku, Sapporo, Hokkaido 063-0052, Japan; Department of Surgery, Doukikai Tomakomai Hospital, 3-9-10 Shin-Nakano-cho, Tomakomai, Hokkaido 053-0006, Japan

**Keywords:** ovarian torsion, omentum, entanglement, elderly, laparotomy

## Abstract

Cord-like omental entanglement is a rare cause of visceral strangulation, previously reported mainly in bowel obstruction. We report a case in which a cord-like segment of omentum wound once around the utero-ovarian ligament, forming a knot that caused right ovarian torsion in an 80-year-old woman. She presented with a progressively enlarging abdominal mass measuring 100 × 220 mm. Laparotomy revealed omental entanglement with 180-degree torsion of the right ovary. Release of the knot restored blood flow, after which right salpingo-oophorectomy and resection of the cord-like omentum were performed. Histopathologic examination confirmed ischemic changes in a pre-existing ovarian cyst consistent with torsion-induced ischemia. The patient was discharged on postoperative Day 23 and remained well at follow-up. This case indicates that a cord-like omentum can act as an extrinsic trigger of ovarian torsion via a novel mechanism, and that ovarian torsion can occur in elderly women despite its predilection for reproductive-age females.

## Introduction

Ovarian torsion is a gynecologic emergency with incompletely understood causative mechanisms. It accounts for 2%–3% of acute gynecologic presentations and occurs most commonly in women aged 29–34 years [1]. The condition typically arises when an ovarian mass increases susceptibility to rotation around the utero-ovarian and infundibulopelvic ligaments [1]. Entanglement of the omentum around the utero-ovarian ligament has not been described previously as a causative mechanism.

Ovarian torsion in postmenopausal or elderly women is uncommon and diagnostically challenging, as the classic presentation of acute pelvic pain may be atypical and an underlying ovarian mass is not always identified [2–4]. Awareness of atypical triggers is therefore essential to avoid delayed diagnosis in this population.

We report a case of right ovarian torsion in an octogenarian woman caused by cord-like omental entanglement around the utero-ovarian ligament.

## Case report

An 80-year-old woman with a history of appendectomy during adolescence and transvaginal myomectomy in her 40s presented to an outside hospital with a 1-day history of vomiting, fever, and urinary retention. On examination, a firm, smooth-surfaced abdominal mass was palpated in the left flank. Vital signs on presentation were: temperature 36.5°C, blood pressure 108/60 mmHg, heart rate 60 bpm, and oxygen saturation 96%.

Plain computed tomography (CT) revealed a unilocular cystic mass measuring 100 × 220 mm without internal septa (Fig. 1). An ovarian cyst was suspected as the most likely diagnosis, although malignancy could not be excluded preoperatively. The mass enlarged progressively over the following 24 h with mild tenderness, and the patient was transferred to our institution for further evaluation. She was admitted 7 days after transfer for preoperative optimization given her multiple comorbidities.

**Figure 1:**
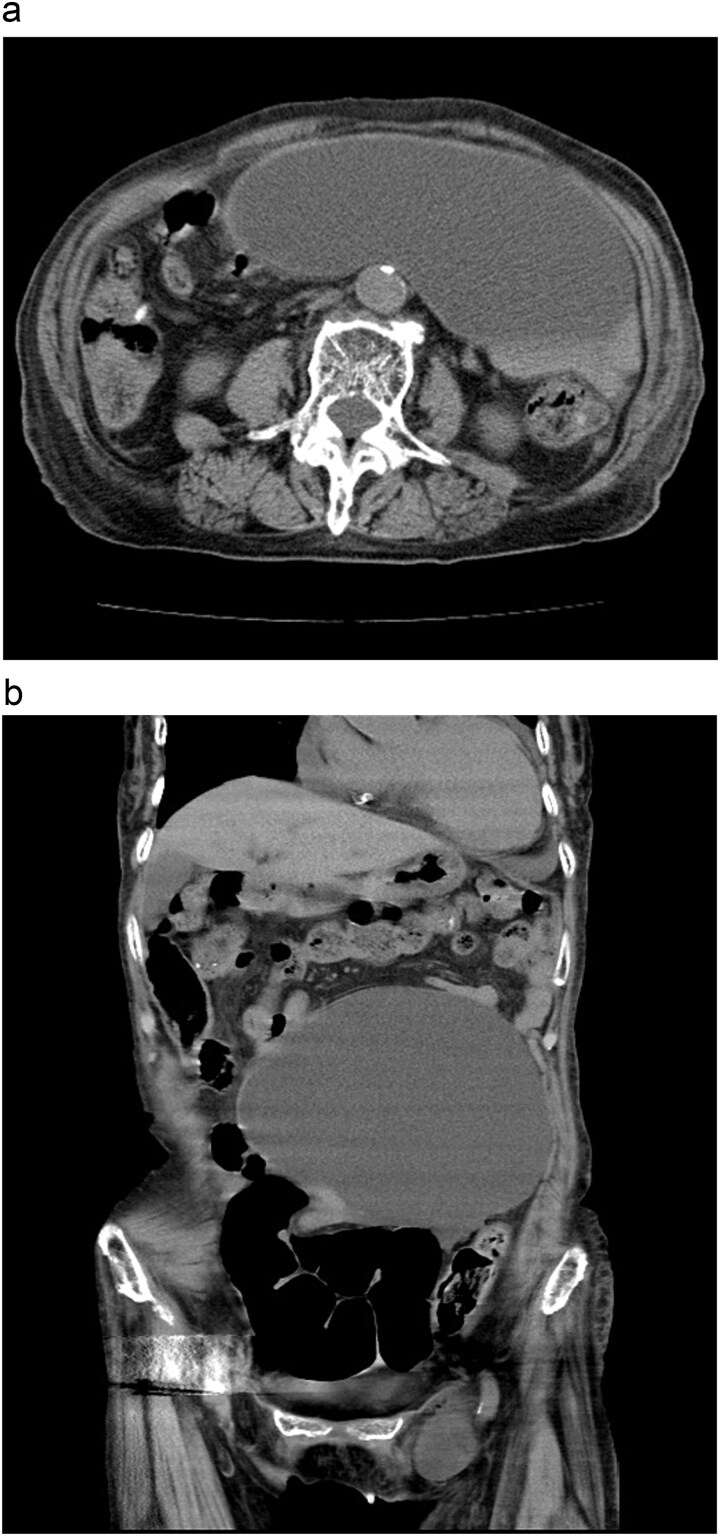
Preoperative unenhanced CT. (a) Axial view showing a large unilocular cystic mass with homogeneous low attenuation and no internal septa. (b) Coronal view demonstrating the enormous size of the cyst occupying the abdominal cavity, with slight leftward displacement despite its right ovarian origin.

Laparotomy was performed at our institution 10 days after initial presentation. Intraoperative findings revealed a cord-like omentum (length 300 mm, diameter 10 mm) extending from the greater omentum that had wound once around the utero-ovarian ligament, forming a firm knot (Figs 2 and 3). The right ovary was twisted 180 degrees in a counterclockwise direction. Upon release of the knot, blood flow to the right adnexa was immediately restored and the torsion resolved, confirming that the omental entanglement was the direct cause of torsion. The right adnexa harbored a pre-existing ovarian cyst measuring 100 × 220 mm with marked torsion-induced congestion; given its size, the mass extended across the midline and was palpable in the left flank despite its right ovarian origin. To exclude malignancy prior to definitive resection, intraoperative aspiration of the cyst fluid was performed; cytologic examination demonstrated reactive mesothelial cells without malignant cells (Class II). The cord-like omentum was resected, and right salpingo-oophorectomy was performed. The operative time was 35 min, and intraoperative blood loss was 10 ml.

**Figure 2:**
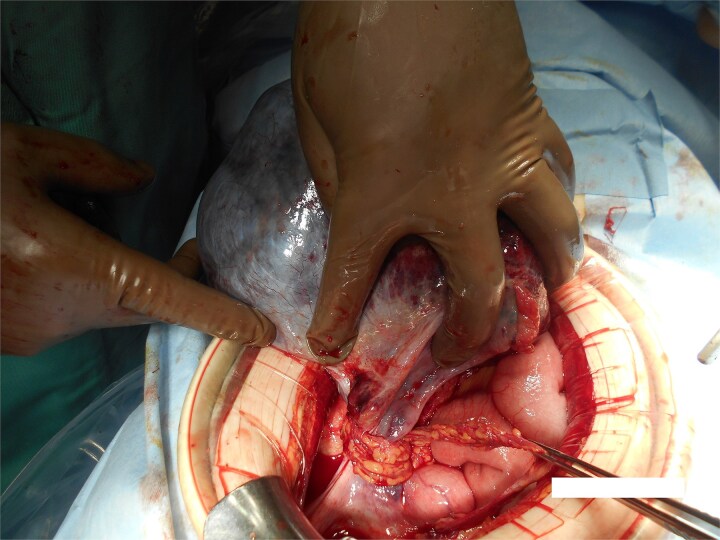
Intraoperative photograph showing the congested right ovarian cyst (upper left) with the cord-like omentum and knot visible at the base of the cyst. The bowel is visible in the lower right.

**Figure 3:**
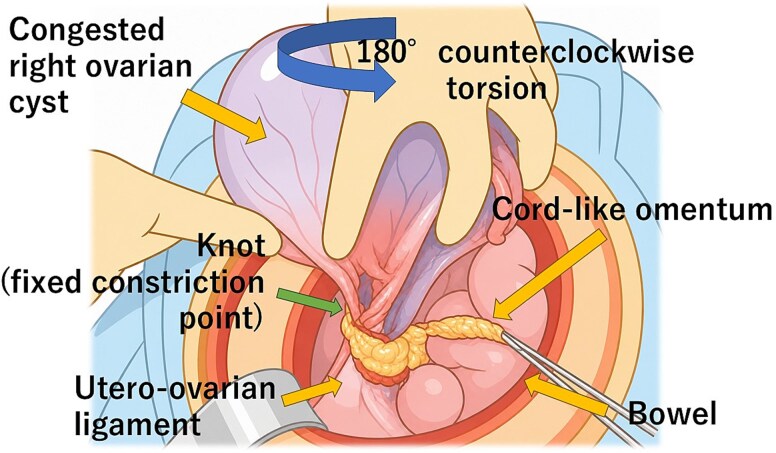
Schematic diagram corresponding to Fig. 2, illustrating the mechanism of omental entanglement. The cord-like omentum wound once around the utero-ovarian ligament and formed a fixed constriction point acting as a fulcrum around which the right ovary rotated 180 degrees counterclockwise.

Histopathologic examination of the resected specimens revealed a dark, hemorrhagic cystic structure (130 × 100 × 20 mm, 78 g) with severe congestion and absence of an epithelial lining, consistent with ischemic changes of a pre-existing ovarian cyst caused by torsion (Fig. 4). The fallopian tube and broad ligament were adherent to the outer cyst wall, further corroborating the diagnosis of torsion-induced ischemia. The resected cord-like omentum measured ~300 mm in length and demonstrated an elongated, fibrous morphology (Fig. 4).

**Figure 4:**
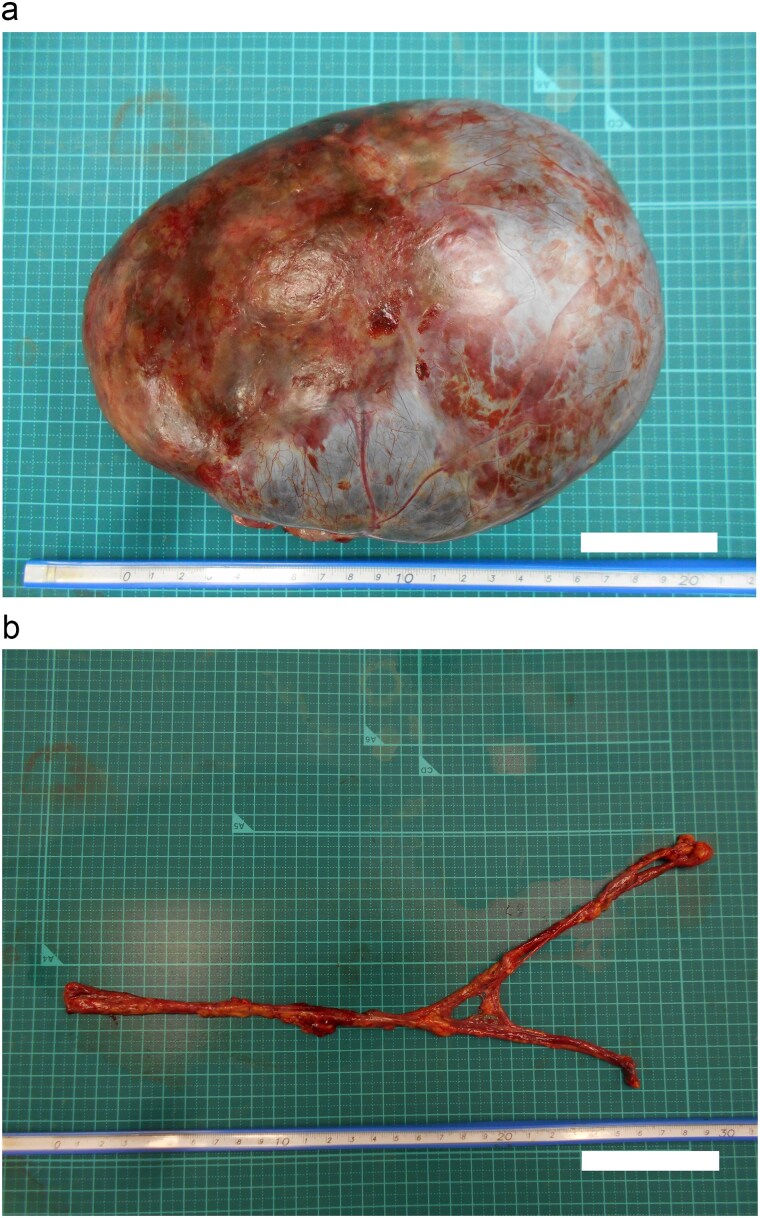
Gross appearance of the resected specimens. (a) The resected right adnexa (130 × 100 × 20 mm, 78 g) showing a dark hemorrhagic cystic surface with severe congestion consistent with torsion-induced ischemia. (b) The resected cord-like omentum (approximately 300 mm in length) demonstrating its elongated, fibrous morphology, which had encircled the utero-ovarian ligament and formed a knot.

The patient resumed oral intake on postoperative Day 1 and was discharged on postoperative Day 23 without complications. At 1-month follow-up, no apparent postoperative complications were identified, and the patient was discharged from outpatient care.

## Discussion

The present case has two notable clinical features: cord-like omental entanglement with knot formation around the utero-ovarian ligament as a novel trigger of ovarian torsion, and its occurrence in an octogenarian woman.

Omental bands have been reported to encircle abdominal viscera such as the small bowel, causing obstruction or strangulation [5–7]. However, to our knowledge, omental entanglement around the utero-ovarian ligament forming a knot and thereby triggering ovarian torsion has not been described previously. Unlike primary omental torsion—in which the omentum rotates on its own vascular pedicle—or conventional ovarian torsion—in which an ovarian mass acts as a lead point—the present mechanism involved an extrinsic cord-like omentum acting as a fixed constriction point around the utero-ovarian ligament, creating a fulcrum around which the right ovary rotated and preventing spontaneous detorsion. The intraoperative finding that release of the knot immediately restored blood flow and resolved the torsion confirmed this causal relationship. The cord-like morphology of the omentum may have resulted from adhesion formation following the patient’s prior appendectomy or transvaginal myomectomy, both of which may cause peritoneal adhesions, potentially leading to fibrous remodeling of the omentum into a cord-like structure.

Ovarian torsion in elderly women is rare and often diagnostically delayed because symptoms may be subacute and the index of suspicion is low in this age group [2–4]. The progressively enlarging abdominal mass in our patient, initially suspected to represent an ovarian cyst of uncertain malignant potential, was ultimately attributable to torsion-induced congestion and ischemic changes of a pre-existing ovarian cyst driven by an atypical mechanism. This case raises the possibility that omental entanglement may warrant consideration in the differential diagnosis of atypical abdominal masses in elderly women, even without classic features of ovarian torsion. This report has several limitations. First, the timeline of knot formation—whether it developed acutely or represented a long-standing finding—could not be determined from available findings. Second, tumor markers were not measured preoperatively, which precluded formal malignancy risk stratification. Third, surgery was performed 10 days after initial presentation rather than on an emergent basis; this interval was attributable to the absence of peritonitis, which prompted initial conservative management with antibiotics, and to the need for thorough preoperative risk assessment given the patient’s multiple comorbidities including cardiac disease and chronic kidney disease.

In conclusion, a cord-like omentum can act as an extrinsic trigger of ovarian torsion by encircling the utero-ovarian ligament and forming a knot—a novel mechanism of which surgeons should be aware when evaluating atypical abdominal presentations, particularly in elderly women.

## Reporting guideline

This case report was prepared in accordance with the CARE (CAse REport) guidelines.
